# Supplementary material for: Fluid curtailment during childhood diarrhea: a countdown analysis
Source: BMC Public Health. 2015 Jun 26;15:588. doi: 10.1186/s12889-015-1878-z (PMC4480991; doi:10.1186/s12889-015-1878-z)
Supplement: Additional file 1: — Surveys and question sequences on home case management of diarrhoea. [file 12889_2015_1878_MOESM1_ESM.docx]

Fluid curtailment during childhood diarrhea:

A Countdown analysis

Jamie Perin,[a*]
jperin@jhu.edu

Liliana Carvajal-Velez, [b]
lcarvajal@unicef.org

Emily Carter, [a]
ecarter@jhu.edu

Jennifer Bryce, [a]
jbrycedandy@aol.com

Holly Newby[b]
hnewby@unicef.org

[a] Institute for International Programs, Johns Hopkins Bloomberg School of Public Health, 615 North Wolfe Street, Baltimore, MD, USA 21205.

[b] Division of Data, Research and Policy, Data and Analytics Section UNICEF, UNICEF, 3 UN Plaza, New York, New York, 10017

*Corresponding author.

Running head: Fluid curtailment during diarrhea episodes

Counts:

Abstract (words): 313

Text (words): 4302

Tables: 3

Figures: 2

References: 23

Web annexes:

1. Surveys and question sequences on home case management of diarrhea used in the analysis.

ABSTRACT

Background

The foundation of recommended diarrhea management in young children is increased fluids and continued feeding. This increase in fluids is necessary to replace those lost during diarrhea and ultimately prevent dehydration. There may be an opportunity to prevent deaths in children under five by discouraging the practice of reducing or curtailing fluids during diarrhea episodes across different settings worldwide.

Methods

We quantify and describe the extent of fluid curtailment in children with diarrhea in a selection of countries (Burkina Faso, Democratic Republic of Congo, Ethiopia, Nigeria, Tanzania, and Uganda) with high burden of diarrhea-related mortality with national cross sectional survey data. We examine the practice of fluid curtailment in these countries and its relationship to child and household traits and to characteristics of diarrhea management.

Results

The prevalence of fluid curtailment among children under five with diarrhea is strikingly high in these countries: 55% in Nigeria, 49% in Ethiopia, 44% in Uganda, 37% in Tanzania, 36% in DR Congo and 32% in Burkina Faso. Fluid curtailment is associated with giving less food, potentially worsening the impact of this harmful practice. Children who were reported to have had fluids curtailed during diarrhea episodes were also 3.51 (95% confidence, 2.66 - 4.64) times more likely to be reported to have food withheld ($\alpha=0.05$; $p < 0.001$). Children who received care from non-governmental providers, and those who were breastfed were more likely to have their fluids curtailed, as were children with an unimproved water source. Children of poorer or less educated mothers and those living in rural areas are more likely to have curtailed fluids, compared to children of less poor or more educated mothers, or those living in urban areas.

Conclusions

The harmful practice of curtailing fluids for a child with diarrhea is highly prevalent, representing an increased risk of dehydration and complications due to diarrhea, including death, especially for children in specific subgroups.

BACKGROUND

Diarrhea is one of the major causes of childhood deaths worldwide, and was responsible for about 580,000 child deaths in 2013.[1] Nearly three-quarters of these deaths occurred in the first two years of life, with the highest burden in Southeast Asia and sub-Saharan Africa. Most deaths from diarrhea occur due to dehydration, across all etiologies.[2] The current WHO/UNICEF recommendations for managing diarrhea and preventing dehydration are to increase fluid intake during diarrhea episodes, including the provision of fluids from oral rehydration salt (ORS), among other interventions.[3] Breastfeeding is a particularly important intervention for prevention and management of diarrhea episodes. Recent evidence, albeit based on a few studies with small sample sizes, suggests that *not* breastfeeding greatly increases the risk of both diarrhea incidence and mortality, particularly at younger ages.[4]

Recent modeling efforts have suggested that 95% of diarrhea deaths could be prevented by 2025 if a set of 15 existing, cost-effective interventions were implemented at scale to improve the physical environment, address nutrition, increase coverage with rotavirus vaccine and provide correct treatment.[5] Correct treatment includes administration of ORS solution and zinc, continued feeding during diarrhea episodes, use of probiotics, and antibiotics for dysentery. Providing increased fluids during diarrhea episodes can prevent dehydration, but is not a treatment for dehydration, because dehydration treatment requires ORS.[6]

One practice known to be harmful is curtailing the fluids offered to children with diarrhea, including breastmilk.[7,8] The purpose of this study is to describe the prevalence of fluid curtailment during childhood diarrhea episodes, and to explore associations between this harmful practice and characteristics of the child, family and community and the correct management of diarrhea episodes that may help target behavior change interventions.

Countdown to 2015 for Maternal, Newborn and Child Health (“Countdown”) is a global initiative that tracks progress in achieving high, sustained and equitable coverage for interventions of proven effectiveness in preventing unnecessary deaths among women and children in 75 priority countries.[9] Countdown

commissioned this analysis with the aim of generating new information that can help increase the effectiveness of programs aimed at reducing child deaths from diarrhea.

A recent systematic review found that there have been few previous studies of fluid curtailment.[10] The evidence base is also difficult to summarize succinctly, because a variety of methods have been used, and even among those using population-based surveys, survey questionnaires used to assess fluid curtailment are not standardized. For example, some surveys ask about fluids *offered* to a child with diarrhea, others about fluids *given* to a child or actually ingested, and unfortunately many do not report on actual formulation of the question posed to caregivers. The review concludes that previous research on the extent of fluid curtailment suggests that this harmful practice is highly prevalent in some settings, but that high quality, recent, and comparable data are scarce.

In this study we use data from recent, standardized, nationally-representative household surveys to examine the practice of fluid curtailment during episodes of childhood diarrhea in six African countries that are among those with the highest burden of diarrhea mortality globally.

METHODS

We selected the six African countries identified in 2012 in the Global Action Plan for the Prevention and Control of Pneumonia and Diarrhea (GAPPD)[2,11] as major contributors to the global childhood mortality burden due to pneumonia and diarrhea that had national survey data collected in 2010 or later: Burkina Faso, Ethiopia, United Republic of Tanzania, Uganda, Democratic Republic of the Congo (DRC) and Nigeria. Together these countries account for about 26% of all under-five deaths due to diarrhea in 2013.[1] The first four countries had conducted Demographic and Health Surveys (DHS) since 2010, and the last two had conducted Multiple Indicator Cluster Surveys (MICS) since 2010. More information on these surveys is available in web annex 1.

MICS and DHS both collect data on fluid intake during childhood diarrhea episodes from nationally-representative samples of mothers or caretakers, using a standardized approach.[12] These questions are

addressed to mothers (DHS) or mothers/caretakers (MICS). In MICS, in cases when the mother is deceased or is living elsewhere, questionnaires are administered to primary caregivers. These questions are asked when respondents report that their child under five years of age had diarrhea within the two weeks prior to the interview. The question about how much the child was given to drink during the diarrhea (including breast milk) is asked in a comparable way in both MICS and DHS. This question is included in the women's questionnaire of DHS and in the questionnaire on children under five for MICS. Both surveys classify responses into six categories: nothing, much less than usual, somewhat less, about the same, more, and don't know. The exact wording of the questions in MICS and DHS as well as sequences used to collect this information are available in web annex 1.

We examined the distribution of fluids given during diarrhea using these six categories, while accounting for the stratification and clustering designs for each survey. We then collapsed the responses into two summary categories for further analysis: 1) "fluids curtailed", defined as children for whom caregivers reported that no fluids were given or that the fluids given were either much less than usual or somewhat less than usual; and 2) "fluids not curtailed", defined as children for whom caregivers reported that either the same amount or more fluids were given. We compare these weighted distributions across the six countries.

In order to inform activities related to advocacy and behavior change interventions, we consider a selection of factors possibly related to fluid curtailment with a priori interest. We classify these into two groups: first, those related to diarrhea management, to clarify if fluid curtailment is compounded with other indicators of treatment. Second, in order to identify those most at risk, we examine factors related to each child and describe factors determined for each household.

For associations between fluid curtailment and other diarrhea case management practices, we investigated the relationships between fluid curtailment and how much food was given to the child during diarrhea (food), ORS administration, reported use of antibiotics and antimotilics and care-seeking for the diarrhea episode. Response options for care-seeking were summarized as: 1) no care-seeking outside

the home; 2) care-seeking from government (public) providers; and 3) care-seeking from all other sources, as defined by survey documentation. We would have liked to include the administration of zinc, but this treatment was reported in only 181 of the 12,012 (1.5%) of the childhood diarrhea cases included in the analysis.

For relationships between fluid curtailment and child and household characteristics, child variables included the age and sex of the child, as well as whether the child was still breastfeeding (yes/no) at the time of the survey. Household characteristics included the mother's educational level (none, primary school, secondary school or more), the number of births or parity of the mother, household wealth (socioeconomic quintile from poorest to least poor as defined in the surveys by the presence of country-specific sets of household assets), urban versus rural residence, and whether the household had an improved water source (yes/no).

We described these relationships by country with tabulations using weights determined by each survey's design, and estimate bivariate statistical differences in fluid curtailment between levels of each factor using Pearson's design-adjusted F test, which accounts for survey related variability.[13,14] Prior to testing for a relationship between breastfeeding and fluid curtailment, we tested for an interaction between age and breastfeeding. In the event that this interaction was present, a test for the overall association between breastfeeding and fluid curtailment was not conducted, as the association would depend on age.[15] As these relationships were primarily descriptive, and intended to identify practices and children and household most at risk, as opposed to attribute cause, we did not consider the potential for other confounders.

Finally, we will summarize the results of observed factor relationships with fluid curtailment for each factor of interest, addressing the potential generalizability to other settings. We estimated the relative risk of fluid curtailment in each country, and meta-analyzed the results to estimate an overall comparison of the rates of fluid curtailment between different groups (Figure 2). These relative risks are modeled with restricted maximum likelihood, controlling for the heterogeneity across surveys with random effects.[16,17] Unless

otherwise noted, all descriptions and relationships account for the clustering and stratification in each national survey.

RESULTS

Weighted tabulations of responses to the question on fluid intake during childhood diarrhea episodes are shown in Table 1, by country survey. For all surveys, the largest response category is “the same as usual”, however, the exact value varies from 30% in Nigeria to 44% in Tanzania. There are few missing or “don’t know” responses, with a maximum of 1% in the 2010 survey in Tanzania. The prevalence of any type of fluid curtailment, in descending order of prevalence, is 55% in Nigeria, 49% in Ethiopia, 44% in Uganda, 37% in Tanzania, 36% in DR Congo and 32% in Burkina Faso. The full response distribution is also shown by survey in Figure 1.

Diarrhea Management

The percent of children with diarrhea whose fluids were curtailed is shown in Table 2, weighted by survey design factors and stratified by characteristics of diarrhea management. Data on care-seeking for diarrhea episodes are not available in the MICS surveys in Nigeria (2011) or DRC (2010) used in this analysis, and data on antimotilic use are not available in the Tanzania DHS. In all other cases, bivariate significance is shown for each factor in each country, to determine whether the percent of children with curtailed fluids is associated with other aspects of diarrhea management.

Children with diarrhea whose mothers report curtailing fluids are also more likely to be given less food (Table 2). This relationship is statistically significant at the 0.001 level in each case. The strongest estimated relationship between food and fluid curtailment is in Nigeria, where 79% of children with food curtailed were also given fewer fluids than usual.

Care-seeking for diarrhea was found to be significantly related to fluid curtailment only in Uganda. Pairwise Student’s *t* tests[18] comparing the three levels of care-seeking (none, government sector and all

other) for children with diarrhea in this survey indicate that fluid curtailment in those who did not seek care is different from fluid curtailment in those who sought care from sources not related to the government (p=.002) (results not shown). For the remaining diarrhea management factors (antibiotic use, antimotilic use, and ORS use), we found no significant associations with fluid curtailment practices in Uganda.

In the meta-analyses of diarrhea management practices (Figure 2), giving less food was strongly associated with increased fluid curtailment across countries, with those who did not decrease the amount of food given having an estimated 0.28 (95% confidence 0.215, 0.376) times the risk of fluid curtailment compared to those whose food was reduced. Those who received care from a non-governmental provider were more likely to be given less fluids across countries compared to those who did not receive care (relative risk 1.14, 95% confidence 1.035, 1.256).

Child Characteristics

The descriptive results on the percent of children for whom caregivers report curtailing fluids during diarrhea episodes are presented by child and household characteristics in each country in Table 3. The results show considerable variability, although some patterns are clear. Prior to testing for a relationship between breastfeeding and fluid curtailment, we tested for an interaction between age and breastfeeding. For all countries this interaction was not significant, indicating that breastfeeding is related to fluid curtailment the same across age in all six countries. Breastfeeding was related to rates of fluid curtailment in three countries. Breastfed children were more likely to have their fluids curtailed in Burkina Faso (34% vs 29%, p=.025), Nigeria (58% vs 53%, p=.028) and Tanzania (41% vs 34%, p=.046).

The results show that children under the age of six months are more likely to have their fluids curtailed than older children in all countries but Nigeria, with statistical significance in Tanzania (p<.01), Burkina Faso (p=0.03) and Ethiopia (p<.01). In each of these three countries, the highest fluid curtailment was in the youngest age group, 0-6 months, and the lowest was in the oldest, 24-59 months. No consistent

pattern of associations was found between fluid curtailment and the sex of the child or the total number of children born to the mother. In the meta-analyses of child characteristics, breastfeeding was associated with increased fluid curtailment across countries, with those who were breastfed at more risk of having their fluids curtailed (relative risk 1.09, 95% confidence 1.026, 1.161).

Household Characteristics

Caregivers in rural areas were more likely than caregivers in urban areas to curtail fluids in all countries, but this difference was not significant in Nigeria or Uganda. Estimated differences in rates of fluid curtailment between urban and rural households, in descending order by the magnitude of the difference, are Tanzania (26% and 41%), Ethiopia (38% and 50%), DRC (28% and 39%) and Burkina Faso (23% and 34%).

Relationships between water source and fluid curtailment are mixed. Households with an improved water source had lower rates of fluid curtailment in Tanzania ($p<.01$), however, there was not sufficient evidence to support this relationship in Burkina Faso, DR Congo, or Ethiopia. In Nigeria and Uganda, the estimated difference in fluid curtailment rates by water source was negligible.

Mothers with less education generally reported giving their children less to drink, especially in Nigeria ($p=.04$), Burkina Faso ($p<.01$), and DRC ($p<.01$). The greatest observed difference in rates of fluid curtailment by education is in DRC, followed by Burkina Faso with the smallest difference in Nigeria. Household wealth is significantly related to fluid curtailment in Tanzania ($p=.02$), Burkina Faso ($p<.01$), and DRC ($p<.01$). In all three of these countries, the least poor are substantially less likely to curtail fluids than poorer households, although patterns differ.

In the meta-analyses of household characteristics, residence location was associated with fluid curtailment, with rural households being more likely to curtail fluids than urban households (relative risk

1.28, 95% confidence 1.132, 1.452). Improved water is associated with less fluid curtailment (relative risk 0.90, 95% confidence 0.813, 0.997), and mothers with more education tend to curtail fluids less often (relative risk for primary vs no education, 0.88 (0.813, 0.951); relative risk for secondary vs no education, 0.75 (0.618, 0.904)). Wealth quintile, the final household characteristic considered, is also related to fluid curtailment across countries. Households in the least poor wealth quintile were less likely to curtail fluids than those in the poorest quintile (relative risk 0.75, 95% confidence 0.58, 0.965) (Figure 2).

This study reports on analyses examining fluid curtailment practices by 12 different characteristics of children, their households, and how their diarrhea was managed, for a total of 90 significance tests, including tests from the meta-analyses. Given a type one error rate of 0.05, we would expect to see four or five false positives.[16] We observed 31 statistical relationships between these factors and fluid curtailment during diarrhea.

DISCUSSION

Previous studies have documented that curtailing fluids for children with diarrhea can be a dangerous practice, running counter to current WHO/UNICEF recommendations for the management of childhood diarrhea and potentially increasing the incidence of dehydration. This is the first analysis that uses recent (2010 or 2011), standardized data on fluid intake practices from MICS and DHS surveys to describe fluid curtailment practices in six countries in sub-Saharan Africa with high burden of diarrhea-attributable mortality.

The results show clearly that the curtailment of fluids among children reporting recent diarrhea is a widespread practice in these countries. Between 32% (Burkina Faso) and 55% (Nigeria) of child caregivers report that they reduced the amount of fluid they gave to their child during the most recent diarrhea episode.

Normative standards for correct home management of diarrhea, including those reflected in the WHO/UNICEF guidelines for both the integrated management of childhood illness[19] and the community

case management of childhood illness[20] recommend that caregivers of children presenting with diarrhea be instructed to increase fluids given to the child and to continue feeding the child. Assessments of the implementation of both strategies, however, have shown that these counselling messages are not consistently provided.[21,22] This is reinforced by our finding that in five of the six countries, seeking care from a government provider was not associated with lower levels of fluid curtailment relative to care-seeking from other providers or no care-seeking outside the home. Our results also show that caregivers who report curtailing fluids are also more likely to stop feeding a child during a diarrhea episode. Earlier reports indicate that younger children, who are at greatest risk of death due to diarrhea,[2] are more likely to have fluids curtailed than older children,[10] however, our results regarding age were mixed across countries.

The findings presented here suggest that there is an important missed opportunity to prevent child deaths due to diarrhea by reaching caregivers with effective behavior change messages about the need to increase, not stop or decrease, giving fluids during diarrhea episodes. These messages must be reinforced in health worker training (including community health workers) and supervision, but may also warrant direct behavior change interventions at community level.

The findings also reveal patterns that may be useful in targeting behavior change programs which aim to build capacity at the community and household level. Although there is variation across countries, the results suggest that fluid curtailment is more common among children who live in rural areas, who live in the poorest households, or who have mothers with no education.

Some aspects of fluid curtailment practices were not able to be investigated thoroughly using the available survey data sets. Sample sizes were too small to support definitive analyses in some areas, notably patterns of fluid curtailment by antimotilic practices and treatment with zinc. In addition, the role of local and cultural beliefs and practices in determining caregiver understandings of diarrhea and appropriate responses would require further study using qualitative methodologies.[23] Explanations for the high rates of fluid curtailment and the motivations of caregivers related to childhood diarrhea also remain

unclear, but could be investigated further in future research. Further secondary analysis could also explore the extent to which the findings from these six countries in sub-Saharan Africa are generalizable to other countries and regions.

These results are also likely to be affected by the difficulty of obtaining accurate responses from caregivers about the amount of fluid ingested by a child during a diarrhea episode. First, the recommended treatment for dehydrating diarrhea is ORS, which is administered by mixing the salts with water. Mothers may consider ORS solution as a medicine, rather than a fluid, affecting whether or not they report curtailing or giving increased fluids during the episode. This may explain our failure to find an association between reports of ORS administration and fluid curtailment. Second, although standardized questionnaire wording was used in asking mothers and caregivers about the fluids they gave the child, the term “gave” could have been ambiguous to respondents or the meaning may have shifted after translation into local languages. In addition, the definition of “fluids”, particularly in relation to breastfeeding, may be subject to variable interpretation across countries or languages, or even among responses from the same survey, with the potential for additional error in our estimates of fluid curtailment. However, the standardization of the survey tools plays an important role in minimizing potential biases. For instance, all surveys in this analysis used the same wording (“gave”) in the core question on fluid intake. In addition, the reported prevalence of fluid curtailment is sufficiently high to warrant attention even if some mothers/caretakers interpreted the question differently.

Despite these limitations, then, these results should alert the public health community to this harmful practice and the need to strengthen program strategies. Of particular importance is the need to determine how contacts with the health system can be used effectively to counsel caregivers on the dangers of dehydration together with the need to increase fluids and continue feeding when their child has diarrhea. Continuing analyses by Countdown can examine whether programs are put in place to reduce this harmful practice and can reduce its overall prevalence as well as inequalities among specific subpopulations.

CONCLUSION AND RECOMMENDATION

Diarrhea deaths are preventable. These findings show that the harmful practice of curtailing fluids when a child has diarrhea is highly prevalent, and that current strategies to promote correct management of childhood diarrhea have not been effective to date in changing this behavior. New and reinforced efforts to reach caregivers, including those not currently utilizing public health services, are needed urgently. No one solution will be enough, and efforts to strengthen counseling during sick child contacts must be buttressed by direct efforts to reach mothers and their communities to ensure that dehydration is prevented in children with diarrhea and that they receive appropriate treatment during the diarrhea episode.

ABBREVIATIONS

DHS, Demographic and Health Survey; MICS, Multiple Indicator Cluster Surveys: UNICEF, United Nations Children's Fund; WHO, World Health Organization; ORS, oral rehydration salts; GAPPD, Global Action Plan for the Prevention and Control of Pneumonia and Diarrhea; DRC, Democratic Republic of the Congo.

ETHICAL CONSIDERATIONS

The survey data analyzed in this research was collected and managed by local government entities with technical support from MICS and DHS. In each country, these government entities managed and approved the informed consent process and other ethical issues. Prior to public release, MICS and DHS make their data anonymous. All data used in this research are anonymous and not identifiable, and so we sought no further ethical approval.

COMPETING INTEREST

The authors declare that we have no competing interests.

AVAILABILITY OF SUPPORTING DATA

This research uses national survey data that is publicly available for research collected by MICS (mics.unicef.org) and DHS (http:/www.dhsprogram.com).

AUTHORS' CONTRIBUTION

EC researched the background for diarrhea treatment in young children. JP analyzed survey data and wrote the first draft of the methods and results. HN, JB, and LCV provided interpretation and refinement for issues relating to analysis. JB and HN wrote the first draft of the background with input from EC. JB and HN wrote the initial discussion and conclusions. All authors contributed to editing the final manuscript.

ACKNOWLEDGEMENTS

This work was funded through a sub-grant from the U.S. Fund for UNICEF under the Countdown to 2015 for Maternal, Newborn and Child Survival grant from the Bill & Melinda Gates Foundation. The authors would also like to thank Shane Khan from the MICS team at UNICEF for his insightful input on the interpretation of household survey data.

Table 1. Fluid curtailment during diarrhea from six recent national surveys. A summary of responses to the question, asked about children under five with diarrhea in the last two weeks: how much was [name of child] given to drink during the diarrhea (including breast milk)? Data are shown as a percent of total responses, weighted according to the survey sampling design, with approximate 95% confidence intervals.

	**Burkina Faso 2010**	**DR Congo 2010**	**Ethiopia 2011**	**Nigeria 2011**	**Tanzania 2010**	**Uganda 2011**
N surveyed	15,044	11,093	11,654	26,018	8,023	7,878
N with diarrhoea	2,031	1,894	1,620	3,949	1,015	1,684
Fluids given						
Nothing	1 (0, 1)%	4 (3, 6)%	7 (5, 9)%	3 (2, 4)%	13 (10, 16)%	5 (4, 6)%
Much Less	6 (4, 7)%	12 (10, 15)%	13 (11, 16)%	26 (23, 29)%	5 (3, 7)%	18 (15, 20)%
Somewhat Less	25 (23, 27)%	20 (17,23)%	28 (24, 32)%	26 (24, 28)%	20 (17, 24)%	21 (19, 24)%
The Same	42 (39, 45)%	31 (27,34)%	35 (31, 40)%	30 (28, 33)%	44 (39, 48)%	37 (34, 40)%
More	26(23, 29)%	32 (29, 35)%	15 (12, 19)%	14 (12, 16)%	18 (15, 22)%	18 (16, 21)%
Don’t Know	0 (0, 1)%	1 (1,2)%	0 (0, 1)%	1 (0,2)%	0 (0, 1)%	1 (0,1)%
Missing	0 (-, -)%	0 (0,0)%	0 (0, 2)%	0 (0,0)%	1 (0, 2)%	0 (-, -)%

Table 2. National survey data from six countries stratified by characteristics of diarrhea management. Number and percent of children with fluids curtailed during diarrhea, represented by those answering "nothing", "much less than usual", or "somewhat less than usual" to the question "how much was [name of child] given to drink during the diarrhea (including breast milk)?" Those with significant relationships at α = 0.05 are shown in bold.

	**Burkina Faso**			**DR Congo**			**Ethiopia**			**Nigeria**			**Tanzania**			**Uganda**		
	**n(% curtailed)**		***p***	**n(% curtailed)**		***p***	**n(% curtailed)**		***p***	**n(% curtailed)**		***p***	**n(% curtailed)**		***p***	**n(% curtailed)**		***p***
**Antibiotics**																		
Yes	180	(33%)	0.465	111	(34%)	0.366	137	(54%)	0.245	461	(54%)	0.608	176	(38%)	0.727	230	(43%)	0.663
No	460	(31%)		557	(37%)		725	(48%)		1665	(55%)		198	(36%)		519	(44%)	
**Antimotilics**																		
Yes	9	(33%)	0.899	17	(42%)	0.704	8	(62%)	0.361	211	(54%)	0.704				38	(47%)	0.614
No	631	(32%)		651	(36%)		854	(49%)		1915	(55%)					711	(44%)	
**Food curtailed**																		
Yes	541	(48%)	**<.001**	527	(44%)	**<.001**	743	(67%)	**<.001**	1848	(79%)	**<.001**	279	(55%)	**<.001**	643	(64%)	**<.001**
No	96	(10%)		142	(23%)		122	(17%)		277	(17%)		98	(21%)		107	(15%)	
**Care seeking by type of Provider**																		
None	241	(30%)	0.602				505	(46%)	0.138				125	(36%)	0.879	149	(37%)	**0.014**
Government Sector	271	(33%)					252	(56%)					112	(38%)		240	(42%)	
All Other	129	(31%)					108	(47%)					140	(38%)		361	(48%)	
**ORS**																		
Yes	135	(30%)	0.583	200	(40%)	0.183	271	(54%)	0.127	461	(58%)	0.314	154	(35%)	0.355	354	(44%)	0.871
No	501	(32%)		467	(35%)		587	(47%)		1656	(54%)		216	(39%)		390	(44%)	
**Total**	637	(32%)		667	(36%)		858	(49%)		2117	(55%)		370	(37%)		744	(44%)	

Table 3. National survey data from six countries stratified by child and household characteristics. Number and percent of children with fluids curtailed during diarrhea, represented by those answering “nothing”, “much less than usual”, or “somewhat less than usual” to the question “how much was [name of child] given to drink during the diarrhea (including breast milk)?” Significant relationships are shown in bold.

	Burkina Faso			DR Congo			Ethiopia			Nigeria			Tanzania			Uganda		
	**n(% curtailed)**		***p***	**n(% curtailed)**		***p***	**n(% curtailed)**		***p***	**n(% curtailed)**		***p***	**n(% curtailed)**		***p***	**n(% curtailed)**		***p***
**Breastfeeding**																		
Yes	236	(34%)	**0.025**	370	(39%)	0.123	538	(50%)	0.245	1010	(58%)	**0.028**	218	(41%)	**0.046**	370	(43%)	0.390
No	405	(29%)		285	(34%)		327	(45%)		1093	(53%)		159	(34%)		380	(45%)	
**Age**																		
0-6m	69	(37%)	**0.033**	76	(42%)	0.431	121	(64%)	**0.004**	249	(55%)	0.985	60	(62%)	**<.001**	69	(39%)	0.336
7-23m	319	(33%)		326	(36%)		365	(48%)		864	(55%)		191	(37%)		384	(46%)	
24-59m	253	(29%)		268	(36%)		379	(45%)		1021	(55%)		126	(31%)		297	(43%)	
**Sex**																		
Boy	318	(31%)	0.836	329	(36%)	0.543	451	(49%)	0.712	1131	(55%)	0.731	195	(37%)	0.689	373	(43%)	0.710
Girl	323	(32%)		341	(37%)		414	(48%)		1003	(55%)		182	(38%)		377	(44%)	
**Parity**																		
1	115	(33%)	0.200	72	(36%)	0.675	105	(43%)	0.175	209	(58%)	0.227	50	(31%)	0.208	90	(39%)	0.529
2	97	(27%)		112	(36%)		141	(42%)		272	(55%)		71	(35%)		120	(42%)	
3	97	(31%)		100	(40%)		136	(51%)		298	(55%)		73	(43%)		126	(47%)	
4+	332	(33%)		366	(35%)		483	(51%)		1282	(55%)		183	(38%)		414	(45%)	
**Household Characteristics**																		
**Area**																		
Urban	117	(23%)	**0.001**	199	(28%)	**0.001**	103	(38%)	**0.029**	297	(51%)	0.222	62	(26%)	**0.002**	130	(39%)	0.254
Rural	524	(34%)		471	(39%)		762	(50%)		1837	(56%)		315	(41%)		620	(45%)	
**Improved water source**																		
Yes	482	(31%)	0.316	288	(33%)	0.065	376	(44%)	0.112	965	(55%)	0.882	174	(31%)	**0.003**	556	(44%)	0.852
No	159	(34%)		382	(39%)		489	(52%)		1169	(55%)		203	(43%)		194	(44%)	
**Mother's education**																		
None	533	(33%)	**0.002**	205	(45%)	**<.001**	623	(51%)	0.278	1403	(58%)	**0.043**	98	(41%)	0.212	127	(45%)	0.499
Primary	84	(27%)		301	(37%)		216	(44%)		365	(51%)		249	(37%)		486	(44%)	
Secondary+	24	(18%)		164	(28%)		26	(39%)		364	(52%)		30	(28%)		137	(41%)	
**Wealth quintile**																		
Q1 (Poorest)	110	(31%)	**0.002**	194	(48%)	**<.001**	319	(51%)	0.556	827	(55%)	0.393	86	(43%)	**0.017**	212	(40%)	0.060
Q2 …	135	(35%)		114	(35%)		139	(47%)		641	(59%)		91	(44%)		182	(50%)	

Q3 …	154	(38%)	151	(39%)	145	(54%)	343	(55%)	83	(40%)	129	(45%)
Q4 …	155	(32%)	146	(33%)	152	(44%)	200	(50%)	79	(32%)	109	(45%)
Q5 (Least Poor)	87	(22%)	65	(21%)	110	(46%)	123	(52%)	38	(25%)	118	(39%)
**Total**	637	(32%)	667	(36%)	858	(49%)	2117	(55%)	370	(37%)	744	(44%)

Figure 1. A summary of responses to the question, how much was child given to drink during the diarrhea (including breast milk)? This was asked of children under five who had diarrhea in the last two weeks, in national surveys (DHS or MICS) from six countries in 2010 or 2011. Shaded bars identify responses corresponding to less fluids given than usual. Data are shown as a percent of total responses, weighted according to the survey sampling design.

Figure 2. A summary of the meta-analyses for the association of diarrhea case management practices and child and household characteristics with fluid curtailment. Relative risk was estimated for each of six countries accounting for survey design, and the overall relative risk across countries estimated with restricted maximum likelihood. Point estimates and approximate 95% confidence intervals are shown for each comparison to a reference factor level, with a solid vertical line at neutrality or no association.

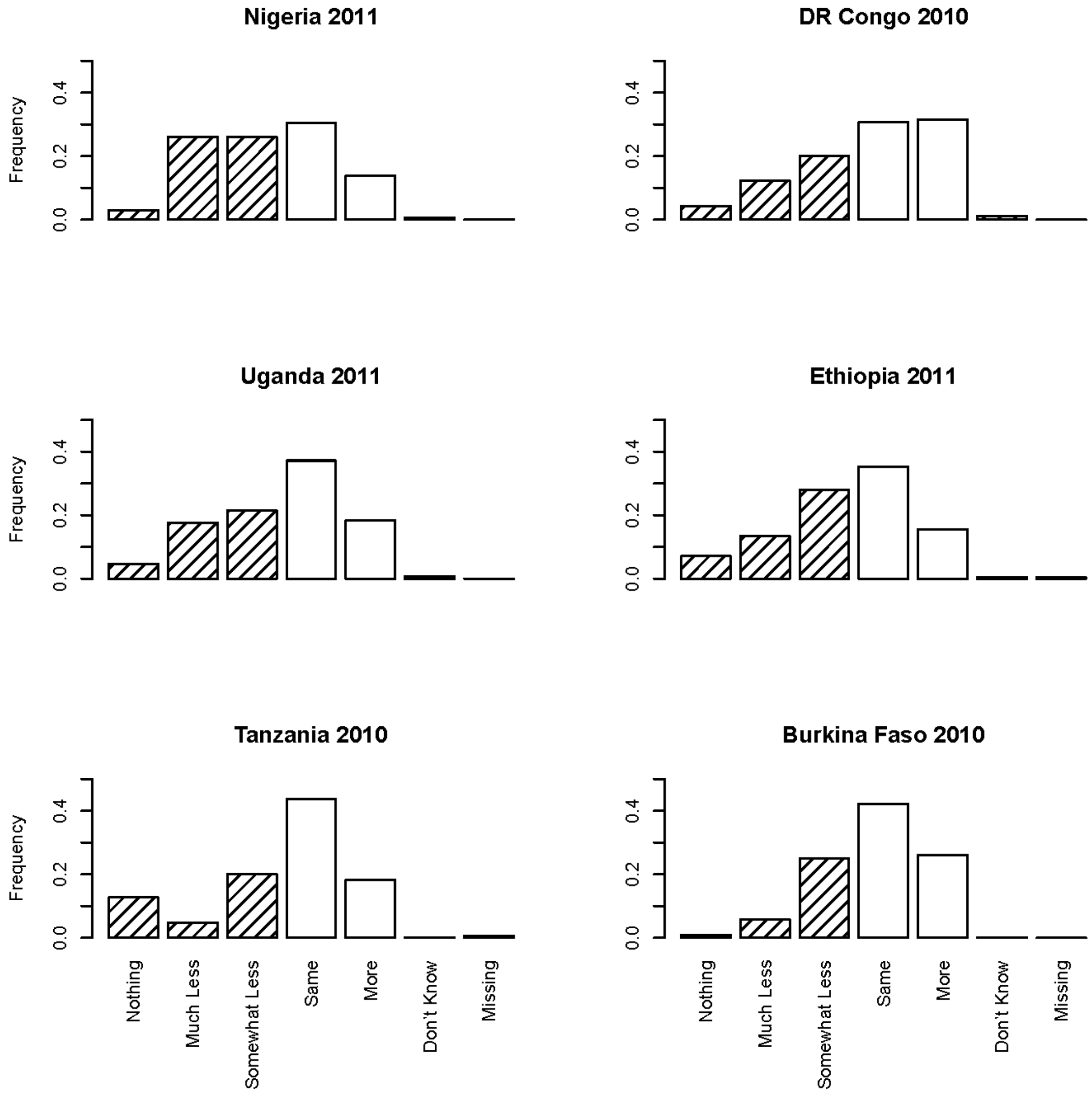
Nigeria 2011
DR Congo 2010
Uganda 2011
Ethiopia 2011
Tanzania 2010
Burkina Faso 2010
Frequency
0.0
0.2
0.4
Nothing
Much Less
Somewhat Less
Same
More
Don't Know
Missing

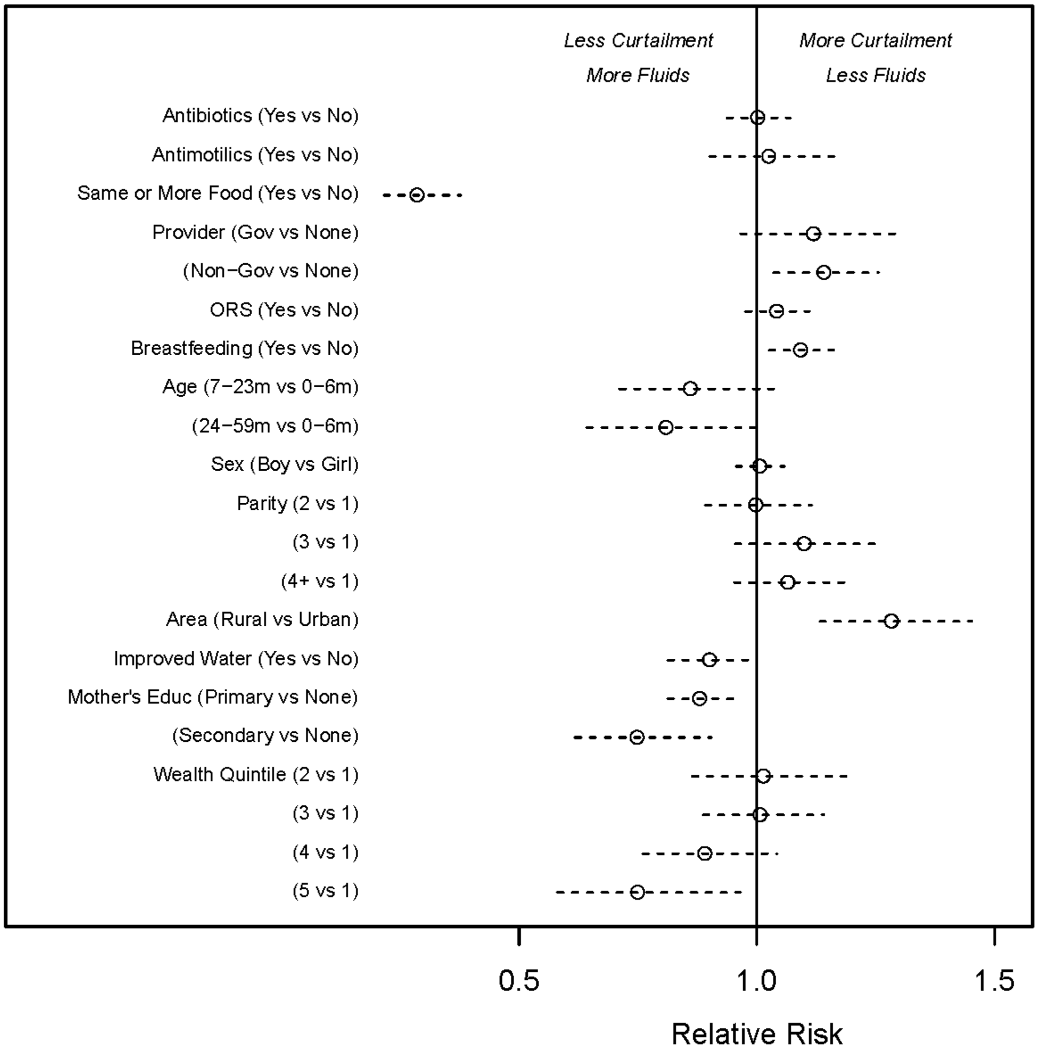

REFERENCES

1 UNICEF, 2014. **Committing to Child Survival: A Promise Renewed**. Progress Report 2014. UNICEF analysis based on IGME 2014, drawing on provisional analyses by WHO and CHERG 2014

2 Fischer Walker CL, Rudan I, Liu L, et al. **Global burden of childhood pneumonia and diarrhea**. *Lancet* 2013, **381**(9875): 1405-1416

3 WHO/UNICEF Joint Statement: **Clinical management of acute diarrhea**, UNICEF, New York, 2004 http://whqlibdoc.who.int/hq/2004/WHO_FCH_CAH_04.7.pdf

4 Lamberti L, Walker CF, Noiman A, Victora C, Black R. **Breastfeeding and the risk for diarrhea morbidity and mortality**. *BMC Public Health* 2011, **11** (suppl 3): S15

5 Bhutta ZA, Das JK, Walker N, et al. **Diarrhea and Pneumonia Interventions Study Group. Interventions to address deaths from pneumonia and diarrhea equitably: what works and at what costs?** *Lancet* 2013; **381**: 1417-29

6 Fischer Walker CL, Fontaine O, Black RE. **Measuring coverage in MNCH: Current indicators for measuring coverage of diarrhea treatment interventions and opportunities for improvement**. *PLoS Medicine* 2013, **10**(5): e1001385

7 Langsten R, Hill K. **Treatment of childhood diarrhea in rural Egypt**. *Social Science & Medicine* 1995, **40**(7):989-1001

8 Babaniyi OA, Maciak BJ, Wambai Z. **Management of diarrhea at the household level: a population-based survey in Suleja, Nigeria**. *East African Medical Journal* 1994, **71**(8):531-5

9 Countdown to 2015 for Maternal, Newborn and Child Survival. Available at http://www.countdown2015mnch.org/, accessed 13 November 2013

10 Carter, E. and others. **Harmful Practices in the Management of Childhood Diarrhea in Low and Middle Income Countries: A Systematic Review**. Manuscript in preparation.

11 World Health Organization/The United Nations Children's Fund (UNICEF). **Ending preventable child deaths from pneumonia and diarrhea by 2025: The integrated Global Action Plan for Pneumonia and Diarrhea**. Geneva, CH: World Health Organization 2013

12 Hancioglu A, Arnold F. **Measuring Coverage in MNCH: Tracking Progress in Health for Women and Children Using DHS and MICS Household Surveys**. *PLOS Medicine* 2013, **10**(5): e1001391

13 Koch GG, Freeman Jr. DH, and Freeman JL. **Strategies in the multivariate analysis of data from complex surveys**. *International Statistical Review* 1975, **43**: 59-78

14 Rao JNK, and Scott AJ. **On chi-squared tests for multiway contingency tables with cell proportions estimated from survey data**. *Annals of Statistics* 1984, **12**: 46-60

15 Korn EL, and Graubard BI. **Simultaneous testing of regression coefficients with complex survey data: Use of Bonferroni t statistics**. *American Statistician* 1990, **44**: 270-276

16 Jackson D, White IR, and Thompson SG. 2010. **Extending DerSimonian and Laird's methodology to perform multivariate random effects meta-analyses**. *Statistics in medicine* 2010, **29**(12):1282-1297

17 DerSimonian R, and Laird N. **Meta-analysis in clinical trials**. *Controlled clinical trials* 1986, **7**(3):177-188

18 Bland, J. Martin, and Douglas G. Altman. **Multiple significance tests: the Bonferroni method**. *British Medical Journal* 1995, **310**(6973):170

18 WHO and UNICEF**. IMCI chart booklet – standard**. 2008. Geneva: World Health Organization.

20 WHO and UNICEF. WHO/UNICEF **Joint Statement on Integrated Community Case Management**. New York: UNICEF, 2012

21 Bryce J, Victora CG, Habicht J-P, Black RE, Scherpbier RW on behalf of the MCE-IMCI Technical Advisors. **Programmatic pathways to child survival: Results of a multi-country evaluation of integrated management of childhood illness**. *Health Policy and Planning* 2005, **20**(S1):i5-i17

22 Gilroy KE, Callaghan-Koru JA,Cardemil CV, Nsona H, Amouzou A, Mtimuni A, Daelmans B, Mgalula L, Bryce J on behalf of the CCM-Malawi Quality of Care Working Group. **Quality of sick child care delivered by Health Surveillance Assistants in Malawi**. *Health Policy and Planning* 2012, **28**(6):573-585

23 Colvin C, Smith H, Swartz A, Ahs J, de Heer J, Opiyo N, Kim J, Marraccini T, & George, A. **Understanding careseeking for child illness in sub-Saharan Africa: A systematic review and conceptual framework based on qualitative research of household recognition and response to child diarrhea, pneumonia and malaria**. *Social Science & Medicine* 2013, **86**: 66-78
